# Supplementary material for: A Joint Model Based on Post-Treatment Longitudinal Prognostic Nutritional Index to Predict Survival in Nasopharyngeal Carcinoma
Source: Cancers (Basel). 2024 Mar 3;16(5):1037. doi: 10.3390/cancers16051037 (PMC10930547; doi:10.3390/cancers16051037)
Supplement: Supplementary file 1 [file cancers-16-01037-s001.zip › cancers-2869182-SI.pdf]

Table S1. Joint modelling analysis of longitudinal PNI data and distant metastasis-free survival (DMFS).

Variable	Estimate (95% CI)	SE	p-value
**Longitudinal sub-model**			
Intercept	44.248 (41.739, 46.757)	1.280	<0.001
Follow-up (years)	-0.822 (-1.038, -0.606)	0.110	<0.001
Age (years)	-0.131 (-0.154, -0.108)	0.012	<0.001
Male	0.540 (-0.071, 1.150)	0.312	0.083
DM	-2.242 (-3.587, -0.896)	0.687	0.001
HTN	0.373 (-0.649, 1.395)	0.522	0.475
BMI (kg/m2)	0.114 (0.050, 0.179)	0.033	0.001
AJCC stage			
1 & 2	Ref.		
3	-0.760 (-1.484, -0.035)	0.370	0.040
4	-2.088 (-2.770, -1.406)	0.348	<0.001
Treatment protocol			
IMRT	Ref.		
CCRT	2.153 (0.833, 3.474)	0.674	0.001
Induction C/T + CCRT	3.608 (2.174, 5.042)	0.732	<0.001
**Variable**	**Hazard Ratio (95% CI)**	**SE**	**p-value**
**Survival sub-model**			
PNI	1.051 (0.970, 1.138)	0.041	0.227
Age (years)	1.011 (0.988, 1.033)	0.011	0.350
Male	2.098 (1.063, 4.144)	0.347	0.033
DM	3.235 (1.116, 9.374)	0.543	0.031
HTN	0.381 (0.116, 1.246)	0.605	0.111
BMI (kg/m2)	0.962 (0.904, 1.023)	0.031	0.211
AJCC stage			
1 & 2	Ref.		
3	6.731 (1.841, 24.611)	0.662	0.004
4	12.958 (3.646, 46.052)	0.647	<0.001
Treatment protocol			
IMRT	Ref.		
CCRT	0.758 (0.235, 2.442)	0.597	0.642
Induction C/T + CCRT	0.532 (0.137, 2.060)	0.691	0.361

PNI = prognostic nutritional index; SE = standard errors; DM = diabetes mellitus; HTN = hypertension; BMI = body mass index; AJCC = *American Joint Committee on Cancer Staging Manual, 7th&8th Edition*; IMRT = intensity-modulated radiotherapy; CCRT = concurrent chemoradiotherapy; C/T = chemotherapy.

Table S2. Joint modelling analysis of longitudinal PNI data and local–regional recurrence-free survival (LRRFS)

Variable	Estimate (95% CI)	SE	p-value
**Longitudinal sub-model**			
Intercept	43.699 (41.165, 46.233)	1.293	<0.001
Follow-up (years)	-1.110 (-1.342, -0.878)	0.118	<0.001
Age (years)	-0.125 (-0.148, -0.102)	0.012	<0.001
Male	0.606 (-0.011, 1.223)	0.315	0.054
DM	-2.392 (-3.749, -1.035)	0.692	0.001
HTN	0.352 (-0.687, 1.390)	0.530	0.507
BMI (kg/m2)	0.128 (0.062, 0.194)	0.034	<0.001
AJCC stage			
1 & 2	Ref.		
3	-0.702 (-1.434, 0.030)	0.374	0.060
4	-1.926 (-2.615, -1.236)	0.352	<0.001
Treatment protocol			
IMRT	Ref.		
CCRT	2.029 (0.687, 3.370)	0.684	0.003
Induction C/T + CCRT	3.529 (2.074, 4.984)	0.742	<0.001
**Variable**	**Hazard Ratio (95% CI)**	**SE**	**p-value**
**Survival sub-model**			
PNI	1.040 (0.956, 1.130)	0.043	0.363
Age (years)	1.018 (0.992, 1.045)	0.013	0.181
Male	2.151 (0.985, 4.697)	0.398	0.055
DM	0.603 (0.133, 2.733)	0.771	0.511
HTN	2.208 (0.940, 5.188)	0.436	0.069
BMI (kg/m2)	0.962 (0.892, 1.037)	0.038	0.308
AJCC stage			
1 & 2	Ref.		
3	1.364 (0.666, 2.794)	0.366	0.396
4	0.960 (0.455, 2.029)	0.382	0.916
Treatment protocol			
IMRT	Ref.		
CCRT	1.077 (0.231, 5.014)	0.785	0.925
Induction C/T + CCRT	0.816 (0.146, 4.554)	0.877	0.817

PNI = prognostic nutritional index; SE = standard errors; DM = diabetes mellitus; HTN = hypertension; BMI = body mass index; AJCC = *American Joint Committee on Cancer Staging Manual, 7th&8th Edition*; IMRT = intensity-modulated radiotherapy; CCRT = concurrent chemoradiotherapy; C/T = chemotherapy.
